# Fentes labiopalatines dans la province du Katanga en République Démocratique du Congo: Aspects épidémiologiques, anatomocliniques et thérapeutiques

**DOI:** 10.11604/pamj.2014.17.319.4268

**Published:** 2014-04-28

**Authors:** Cedrick Milindi Sangwa, Olivier Mukuku, Christian Tshisuz, Jules Mulefu Panda, Mireille Kakinga, Marius Feruzi Kitembo, Jean-Felix Mutomb, Bwana Fwamba Odimba

**Affiliations:** 1 Faculté de Médecine, Université de Lubumbashi, Lubumbashi, République Démocratique du Congo

**Keywords:** Fente labiopalatine, épidémiologie, anatomoclinique, prise en charge, Katanga, Cleft lip palate, epidemiology, clinicopathology, management, Katanga

## Abstract

Les fentes labiopalatines sont les malformations les plus rencontrées de la sphère orofaciale. L'objectif est de décrire le profil épidémiologique, anatomoclinique et thérapeutique des fentes labiopalatines observées dans la province minière du Katanga au sud-est de la République Démocratique du Congo. Il s'agit d'une étude transversale réalisée dans quatre institutions hospitalières de la province du Katanga dans des districts sanitaires différents (Hôpital Jason Sendwe à Lubumbashi, Hôpital Gécamines Panda à Likasi, Hôpital Gécamines du personnel à Kolwezi, Hôpital General de référence de Kamina) et qui a porté sur 154 cas de fentes labiopalatines enregistrés au cours de la période allant du 1er mai 2010 au 30 septembre 2012. L'âge moyen de consultation était de 11,8 ans et une prédominance masculine (55,2%) était notée. Un pic était noté chez les deux premiers nés de la famille (55,8%). Nous avons enregistré 20,7% des cas de consanguinité dont 54,2% de premier degré. La fréquence des différents types de fentes labiopalatines diminue au fur à mesure que la fente s'étend de la lèvre supérieure au palais en passant par l'alvéole : 72% (labiales), 21,4% (labiopalatines) et 7,7% (palatines). Les variétés unilatérales sont plus fréquentes (76,7%) que les bilatérales (16,1%). Dans les formes unilatérales, le côté gauche est plus concerné (47,1%) par rapport au côté droit (38,6%). L'évaluation de la gravité selon Anastassov montre que 50,6% de nos patients étaient de degré moyen et 16,2% étaient à un degré sévère. Les malformations associées ont été retrouvées dans 5% des cas et elles sont à prédominance squelettique. C'est la technique de Millard qui a été la plus pratiquée (72/130). Les résultats étaient excellents dans 71,5% contre 1,5% de mauvais. Le séjour d'hospitalisation était de 3 jours et le taux de complications post opératoire était de 2,98%.

## Introduction

Parmi les anomalies congénitales de la face, les fentes labiopalatines (FLP) sont de loin les plus fréquentes et leur prévalence mondiale va de 0,8 à 2,7 pour 1000 naissances [1]. Cette prévalence varie en fonction des groupes ethniques ; la prévalence la plus basse étant rapportée chez les Afro-américains (environ 0,5%) [2, 3] et les Caucasiens (environ 1%) [1], et la plus élevée chez les natifs américains (environ 3,5%) et les Asiatiques (environ 1,7%) [4–6]. En Afrique subsaharienne, les études menées respectivement au Nigeria et en Ethiopie par Eigbobo et Mekonem rapportent des prévalences respectives de 0,4% et 1,49% [7, 8]. Une étude menée à Lubumbashi sur les malformations congénitales rapporte une fréquence des FLP de 0,65% [9].

Malgré le fait que des multiples causes possibles soient en voie d'investigation, aucune cause unique n'a été mise en évidence. La majorité des fentes semble être due à une combinaison de facteurs héréditaires et environnementaux. D'après une récente étude réalisée par Longombe à l'Est de la République Démocratique du Congo, l'étiologie des FLP serait plus liée aux facteurs environnementaux que génétiques [10].

Un nombre considérable d'études épidémiologiques faites sur les FLP dans les différentes régions du monde, démontrent presque souvent des informations divergentes sur la distribution de ces malformations dans les populations africaines comparativement aux populations occidentales ou asiatiques [11].

Au Katanga en particulier, ce sujet aussi important n'a pas encore été étudié et il faut noter que les enquêtes statistiques sont rendues difficiles par le grand nombre d'accouchement à domicile non répertoriés, notamment dans le monde rural. Ainsi aucune donnée statistique n'a été encore publiée à ce jour raison pour laquelle, nous avons mené cette étude ayant pour objectif de décrire le profil épidémiologique, anatomoclinique et thérapeutique des FLP rencontrées dans le sud de la République Démocratique du Congo plus précisément dans la province du Katanga.

## Méthodes

Ce travail a été réalisé dans les hôpitaux généraux de 4 villes de la province du Katanga (Sud de la République Démocratique du Congo) : Lubumbashi, Likasi, Kolwezi et Kamina. Cette province est caractérisée, ses dernières années, par une augmentation des activités minières sans le strict respect de l'application des normes de sécurité environnementale (exploitation manuelle et artisanale des minerais, stockage des minerais dans les maisons d'habitation, transport des minerais à l'air libre, absence d'équipement de protection) [12].

Il s'agit d'une étude transversale qui a été menée pendant la période allant du 1er mai 2010 au 30 septembre 2012 au cours de laquelle nous avions reçu 154 sujets porteurs de FLP lors d'une campagne d'opérations gratuites de FLP organisée par l'organisation non gouvernementale **Smile Train**. Etant donné le niveau de vie bas de la population et que cette pathologie exige des soins spécialisés, les organisateurs ont voulu donné accès aux démunis. L'équipe chirurgicale était composée de deux chirurgiens, d'un anesthésiste et de deux infirmiers et se déplaçait dans chaque ville pendant la période d'étude. Ont été inclus tous les patients ayant été examinés et/ou été opérés pour une FLP par notre équipe dans le service de chirurgie des 4 institutions médicales choisies pendant la campagne allant de mai 2010 à septembre 2012.

Les paramètres étudiés étaient : Les aspects épidémiologiques : âge, sexe, rang familial, antécédent familial de FLP ; Les aspects cliniques : type et variété de la FLP dont le diagnostic clinique s'est basé sur la classification internationale de Kernahan et Stark [13], évaluation de la sévérité initiale de la fente selon le score proposé par Anastassov [14], malformations congénitales associées; Les aspects thérapeutiques et évolutifs : technique opératoire utilisée, type d'anesthésie, évaluation des résultats postopératoires à partir du score élaboré par Anastassov [14], suites postopératoires immédiates, séjour hospitalier.

Concernant les interventions chirurgicales, pour les fentes labiales, nous avons utilisé l'une de 3 techniques suivantes : Veau, Millard, et Tennison ; la technique de Furlow a été utilisée pour les fentes du palais mou et celle de Wardill pour les fentes du palais dur [15]. Les indications opératoires de chaque patient ont été établies par les chirurgiens. La technique chirurgicale était fonction du type de fente. Signalons toute fois que dans notre série, compte tenu du plateau technique à notre disposition, les opérations sur l'arc gingival osseux n'ont pas été faites. L'anesthésie était soit locale, soit générale en fonction de l'âge du patient et du type d'intervention à pratiquer. Nos données ont été saisies et analysées grâce au logiciel Epi info 2011. La moyenne arithmétique, l'écart-type, le médian et le mode ont été calculées à partir des données obtenues.

**Considérations éthiques** : Avant d'être opéré, le patient devait donner son consentement verbal et écrit et pour un patient mineur, c'est son responsable qui devait donner son consentement. Les patients étaient traités par un procédé nécessité par son état de santé. La prise en charge de patients était complètement gratuite.

## Résultats

### Aspects épidémiologiques (Tableau 1)

L'âge moyen était de 11,8 ans avec un écart-type de 11,5 ans (médiane de 9 ans). Le patient le moins âgé avait 3 mois et les plus âgés 54 ans. Soixante-deux virgule trois pourcent de nos patients ont consulté à un âge supérieur à 5 ans. Nous avons constaté une prédominance du sexe masculin avec 85 cas (55,2%) donnant ainsi un sexe ratio de 1,2. S'agissant du degré de consanguinité, nous avons enregistré 6 cas (3,9%) de consanguinité de premier degré et 10 soit 6,5% de deuxième degré. Quant à la position dans la fratrie, plus de la moitié (55,2%) de nos patients occupés les 2 premiers rangs dans la famille.

**Tableau 1:** Répartition des patients selon les aspects épidémiologiques

Paramètre	Effectif (n=154)	Pourcentage
**Age**		
≤5 ans	58	37,7
6-10 ans	29	18,8
11-15 ans	28	18,2
≥16 ans	39	25,3
**Sexe**		
Masculin	85	55,2
Féminin	69	44,8
**Antécédent familial de FLP**		
Absent	130	84,4
Premier degré	13	8,4
Deuxième degré	11	7,2
**Rang familial**		
1	31	20,1
2	55	35,7
3	24	15,6
4	19	12,3
5	17	11,0
≥6	14	9,0

### Aspects cliniques (Tableau 2)

Les fentes du palais primaires (FL et FLA) sont majoritaires soit 71,4%. Nous remarquons que les fentes labiopalatines diminuent au fur et à mesure qu'on s'étend du palais mou vers le palais secondaire en passant par l'alvéole. En ce qui concerne les variétés, nous constatons que les fentes labiales incomplètes sont plus fréquentes (30,8%) que les complètes (10,9%). La fente labiale incomplète gauche est la variété la plus fréquente (22%). Les variétés unilatérales qu'elles soient simple ou totales sont plus fréquentes (76,7 %) que les bilatérales (16,1%). Dans les formes unilatérales, le côté gauche est plus concerné (47,1%) par rapport au côté droit (38,6%). Les fentes labiopalatines totales bilatérales ont été presque exclusivement retrouvées chez les sujets féminins, soit 6 cas sur 7 (85,7%) contre 1 cas sur 7 (14,2%) chez les sujets masculins. La division palatine est moins fréquente (7,7%) et a été trouvée en nombre égale dans les deux sexes.

**Tableau 2:** Répartition des cas selon les aspects cliniques

Paramètre	Effectif (n=154)	Pourcentage
**Type**		
FL incomplète gauche	34	22,0
FL incomplète droite	13	8,4
FL complète Gauche	13	8,4
FL complète droite	4	2,6
FL bilatérale	13	8,4
FLA gauche	14	9,0
FLA droite	14	9,0
FLA bilatérale	5	3,2
FP	12	7,7
FLP gauche	12	7,7
FLP droite	15	9,7
FLP bilatérale	7	4,5
**Degré de sévérité de la fente**		
Moyenne	89	57,7
Modéré	58	37,6
Sévère	5	3,2
Très sévère	2	1,2
**Malformations associées**		
Aucune	146	94,8
Pieds bot	5	3,2
Microcéphalie	1	0,6
Syndactylie	1	0,6
Cardiaque	1	0,6

FL : fente labiale ; FLA : fente labio-alvéolaire ; FP : fente palatine ; FLP : fente labio-palatine

Selon le score de sévérité initiale de la fente, 16,2% (25 patients) étaient à un degré sévère et 8,4% (13 patients) à un degré très sévère. La moitié de nos patients étaient à un degré moyen. Dans notre série, 8 patients soit 5,1% avaient d'autres malformations congénitales associées dont 62,5% de pieds bots.

### Aspects thérapeutiques et évolutifs (Tableau 3)

Des 154 patients examinés, 130 soit 84,4% ont pu être opérés. Les 24 autres patients n'ont pas été opérés suite à des contraintes techniques (manque d'équipement anesthésique et chirurgical). Plus de la moitié (72/130) de patients ont été réparés par la technique de Millard et l'évaluation de nos résultats postopératoires a donné un mauvais score dans seulement dans 1,5% des cas (2/130). Quant aux suites postopératoires immédiates, nous avons noté l'infection chez 2 patients (1,5%) et le lâchage de fil chez un patient (0,7%). Le séjour hospitalier moyen était de 3,4 jours avec un écart-type de 2,1. Le séjour le plus court était de 1 jour et le plus long de 5 jours.

**Tableau 3:** Répartition des patients en fonction des aspects thérapeutiques et évolutifs

Paramètre	Effectif (n=130)	Pourcentage
Anesthésie		
Générale	62	47
Locale	68	52
**Techniques chirurgicales**		
Veau	45	34,6
Tennison	15	11,5
Millard	70	53,8
Résultats		
Excellent	70	53,8
Très bon	22	16,9
Bon	14	10,7
Satisfaisant	18	13,8
Mauvais	6	4,6
**Suites opératoires immédiates**		
Infections	2	1,53
Lâchage de fil	1	0,7
Simples	127	96,9
**Durée d'hospitalisation**		
1 jour	25	19,2
2 jours	14	10,7
3 jours	75	57,6
4 jours	9	6,9
5 jours	7	5,3

## Discussion

L'âge moyen de consultation est de 11,8 ans avec plus de 60% de patients âgés de plus de 5 ans et le patient le plus âgé avait 54 ans. Ceci est en contradiction avec les séries occidentales où la prise en charge des FLP se fait avant l'âge de 6 mois grâce à la recherche systématique des malformations chez le fœtus au cours de la surveillance prénatale mais aussi grâce à la facilité d'accès de la population à une structure spécialisée [16–19]. Dans notre pays, comme dans la plupart des pays en développement, la prise en charge des FLP est très souvent l'apanage des missions humanitaires qui viennent au secours d'une majorité de patients pour qui l'accessibilité aux soins adéquats spécialisés est difficile expliquant ainsi le retard de consultation [7, 10, 20]. En plus de cela, dans les sociétés africaines, l'accouchement d'un enfant malformé est vécu comme un véritable drame compte tenu des considérations mystico-religieuses qui l'entourent. Les enfants porteur de fente labiopalatine à la naissance sont très souvent cachés par leurs familles et peuvent constituer une source de divorce des parents.

Nous avons noté une prédominance masculine avec un sexe ratio de 1,2. Cette prédominance est rapportée par plusieurs auteurs et varie entre 50,5% et 65,8% [8, 10, 21–24]. Comme pour la plupart de malformations congénitales cardiaques, la prédominance masculine est à noter dans les FLP sans aucune explication fournie jusqu'à ce jour. Cependant, d'autres auteurs ont fait un constat différent : c'est le cas de Liluis [25] en Finlande, d'Omo-Aghoja [26] au sud du Nigéria et de Diombana [27] au Mali qui ont trouvé respectivement 50,6%, 51,5% et 53,8% des sujets féminins. Trente-cinq virgule sept pourcent de nos patients occupent la deuxième position dans la fratrie. Ces résultats concordent avec ceux de Lomgombe qui a observé un pic des FLP à la deuxième grossesse (19,1%) [10]. Onyango, dans sa série, rapporte qu'une présence importante de FLP a été retrouvée chez les trois premiers enfants dans la famille. Le rang de naissance semble influencer considérablement la survenue d'une FLP [28].

En recherchant l'antécédent de FLP dans la famille, nous avons noté que 20,7% de nos patients avaient un membre de famille porteur de cette malformation dont 54,2% de premier degré. Cette notion d'antécédent de FLP a été retrouvée chez les patients venus d'une contrée nord-ouest de la province du Katanga où le mariage consanguin est de pratique courante. Près de 4 patients sur 5 n'ont pas présenté d'antécédent familial de FLP dans notre série. Dans l'étude menée à l'est de la RD Congo, seul 3% de patients présentaient cet antécédent hérédo-familial [10]. Nous pensons aussi que dans notre pays, l'étiologie de la FLP serait plus liée aux facteurs environnementaux qu'héréditaires. Schinz, cité par Diombana, pense qu'il serait possible que l'augmentation de la radiation atmosphérique joue un rôle dans la survenue des malformations orofaciales [27]. En effet, il existe de fortes présomptions quant aux effets tératogènes des minerais exploités dans cette province du pays et ces présomptions sont liées aux taux anormalement élevés de ces minerais (Arsenic, Cadmium, Cobalt, Cuivre, Plomb et Uranium) retrouvés dans les urines de la population générale vivant à moins de 3-10 km des zones d'exploitation au Katanga [9, 29].

S'agissant de la forme des fentes, dans notre série, les fentes du palais primaire arrivent en première position 71,4%. Les fentes labiopalatines et les fentes palatines isolées ont représentées respectivement chacune 22% et 7,7%. Nos résultats sont similaires à ceux de Longombe qui a eu 59,6% de fentes labiales isolées, 37,1% de FLP et 2,2% de fentes palatines [10], mais différents de ceux Suleiman [30] au Khartoum qui a trouvé seulement 16% de fentes labiales. Dans son enquête en Ethiopie Eshete [8] a trouvé les FLP majoritaires soit 57,8 %. Dans beaucoup d'études, les fentes palatines sont minoritaires [11]. Nous pensons comme Longombe que l'expression faciale d'une fente palatine isolée entraine moins de traumatisme psychique du fait qu'elle est cachée et est donc mieux supportée que la fente labiale qui elle est bien vue et considérée encore en Afrique comme une honte et une une malédiction pour les parents [10].

Concernant le type de fente, dans notre milieux, nous avons observés que les variétés unilatérales qu'elles soient simples ou totales sont plus fréquentes (77,2%) que les bilatérales (16,2%). Eshete a rapporté la même réalité 57,8% des fentes labiales et/ou palatines unilatérales contre 31,2% des fentes labiales et/ou palatines bilatérales [8]. La fente palatine isolée est encore moins fréquente. Dans les deux séries, elle était respectivement de 7,8% et 9,3%, des taux de loin inférieurs à celui trouvé par Rustermeyer [31] en Allemagne qui est de 45% des cas. Cette divergence pourrait s'expliquer par le fait que contrairement à d'autres cieux, en Afrique, cette malformation cachée n'est pas systématiquement recherchée à la naissance. Un seul cas de fente palatine sous muqueuse a été observé.

Nous avons aussi noté que les FLP incomplètes sont les plus rencontrées avec une fréquence (59,7%) de loin supérieure à celui de Suleiman à Khartoum (Soudan) [30] qui est de 16 % tandis que notre taux des FLP complètes de 21,4% est de loin inférieur à celui du même auteur qui a trouvé 54%. Quant aux fentes bilatérales, nous avons obtenu 16,2% des cas, résultat similaire à celui de Longombe [10] qui les a eut dans 17,6% des cas.

En rapport avec le coté des fentes labiales, plusieurs auteurs ont constatés que les FLP concernent dans la majorité des cas le côté gauche suivi par le côté droit puis viennent les formes bilatérales [8, 10, 11]. Nous avons fait le même constat, car 47,4% des FLP concernaient le côté gauche, 29% localisées à droite et la proportion des fentes bilatérales était de 16,2%. A l'instar d'Eshete [8], nous n'avons observé aucun cas de fente médiane. Signalons toute fois qu'Omo-Aghoja a trouvé une répartition égale des fentes unilatérales à gauche comme à droite [26]. L'étude rapporte 4,5% de malformations associées avec une prédominance des atteintes squelettiques (5 cas) puis cranio-faciales (1 cas) et cardiaques (1 cas). Nos résultats sont superposables à ceux retrouvés dans la littérature où les types de malformations associées les plus fréquentes sont squelettiques, cardiaques et céphaliques [32, 33]. A Stockholm, dans la série de Milerad, les malformations des membres supérieurs et inférieurs et de la colonne vertébrale venaient en première position et représentaient 33% suivi des malformations du système cardiovasculaire 22% [34].

L'évaluation de la gravité selon Anastassov montre que 50,6% de nos patients étaient de degré moyen et 16,2% étaient à un degré sévère. Nos résultats sont différents de ceux obtenus par Mortier [35] qui rapporte 25,6% de patients à un degré modéré et aucun patient à un degré sévère. Ces divergences pourraient s'expliquer tout simplement par la diversité des formes anatomiques des fentes retrouvées dans nos populations respectives.

Des 154 patients de notre série, 130 ont été opéré soit 84,5%. La technique de Millard a été la plus pratiquée soit 55,8% (72/130). Cette technique a été utilisée dans la totalité des cas par Mortier et Anastassov [14, 35]. Et quant aux résultats postopératoires obtenus, dans 71,5% (93/130) des cas le score était excellent. Nos résultats sont comparables à ceux de Mortier et Anastassov qui ont eu respectivement 70% et 64% des résultats excellents [14, 35]. Ces similitudes pourraient s'expliquer par l'utilisation de la même technique opératoire.

Notre étude relève un taux de morbidité de 2,9% fait comparable à ceux de Diakité (2,7%) [32] et de Cronin (3,8%) [36] mais largement inférieur à ceux de Lees (en Angleterre) [37] et d'Abdurrazaq (au Nigeria) [38] qui rapportent respectivement 26,2% et 14,1% de complications. Selon Diah, plusieurs paramètres entrent en jeu pour l'apparition de complications lors de la chirurgie de FLP dont la technique chirurgicale, l'expertise du chirurgien, la largeur de la fente, une mauvaise cicatrisation de la plaie ou une infection du site exploité [39] mais aussi le degré de sévérité de la FLP.

## Conclusion

A l'issue de cette enquête, il se dégage que les aspects épidémio-cliniques de FLP dans la province du Katanga ne sont pas différents de ceux rapportés dans l'Est de la République Démocratique du Congo ni dans d'autres régions de l'Afrique. L'accessibilité difficile de notre population aux soins, le manque d'expertise pour ce genre de chirurgie et l'influence des croyances des populations des milieux surtout ruraux expliquent le retard de consultation.
